# Torque Teno Virus Load Is Associated With Subclinical Alloreactivity in Kidney Transplant Recipients: A Prospective Observational Trial

**DOI:** 10.1097/TP.0000000000003619

**Published:** 2021-08-19

**Authors:** Konstantin Doberer, Frederik Haupenthal, Maja Nackenhorst, Florian Bauernfeind, Florentina Dermuth, Michael Eigenschink, Martin Schiemann, Johannes Kläger, Irene Görzer, Farsad Eskandary, Roman Reindl-Schwaighofer, Željko Kikić, Georg Böhmig, Robert Strassl, Heinz Regele, Elisabeth Puchhammer-Stöckl, Gregor Bond

**Affiliations:** 1 Division of Nephrology and Dialysis, Department of Medicine III, Medical University of Vienna, Vienna, Austria.; 2 Department of Pathology, Medical University of Vienna, Vienna, Austria.; 3 Center for Virology, Medical University of Vienna, Vienna, Austria.; 4 Department of Urology, Medical University of Vienna, Vienna, Austria.; 5 Division of Clinical Virology, Department of Laboratory Medicine, Medical University of Vienna, Vienna, Austria.

## Abstract

Nonpathogenic torque teno viruses (TTVs) are highly prevalent in transplant recipients and associated with immunosuppression. Studies in kidney transplant patients have proposed assessment of TTV load for risk stratification of clinically overt graft rejection. The value of TTV quantification in the context of subclinical rejection has not been evaluated.

**Methods.:**

In this prospective trial, 307 consecutive kidney transplant recipients were subjected to per-protocol monitoring of plasma TTV. TTV was analyzed in the context of protocol biopsies (n = 82), scheduled 1 year posttransplantation.

**Results.:**

TTV load at the time of biopsy was lower in recipients with rejection (n = 19; according to Banff, including borderline changes suspicious for acute T cell-mediated rejection) than those without rejection (n = 63) whereby each log increase in TTV copies/mL decreased the risk for rejection by 9% (risk ratio 0.91, 95% confidence interval, 0.85-0.97; *P* = 0.004). Development of chronic lesions (cg, cv, ci, ct, ah, ptcml) was associated with the number of days with a TTV load <1 × 10^6^ copies/mL between months 3 and 12 posttransplant (β 0.07, 95% confidence interval, 0.01-0.14; *P* = 0.02).

**Conclusions.:**

This trial demonstrates an association between TTV and subclinical graft rejection in kidney transplant recipients. A TTV load <1 × 10^6^ copies/mL suggests suboptimal immunosuppression.

## Abstract 2

Supplemental Digital Content is available in the text.

## INTRODUCTION

Immunosuppression after kidney transplantation is crucial for reducing the risk of organ rejection. Despite this benefit, the compromised immune competence of the recipient leads to an increased risk for infectious disease. Moreover, current immunosuppressive regimens are unable to sufficiently control the allorecognition of the graft in some patients, which may lead to chronic rejection.^1^ Thus, the optimal management of immunosuppressive drug dosing requires a delicate balance between inadequate and excessive immunosuppression. At present, there is no diagnostic test or algorithm available for the optimal guidance of immunosuppressive drugs in the clinical routine.^2^ Monitoring is mainly based on the quantification of the calcineurin inhibitor trough level in the peripheral blood, which correlates more closely with the risk of drug-related toxicity than with effectiveness of the immunosuppression.^3^

Monitoring the torque teno virus (TTV) is a promising new strategy for quantifying the immune function. TTV can be detected in up to 90% of healthy individuals and has not been linked to any human disease.^4^ The prevalence of TTV in immunocompromised patients after transplantation is up to 100% and the virus is unaffected by conventional antiviral drug therapy used in the posttransplantation setting.^4^ The TTV load is directly associated with the amount and type of immunosuppressive drugs administered to the transplant recipient and is thus indirectly associated with graft rejection and infectious disease.^4^ In kidney transplant recipients, recent data suggest TTV quantification for risk stratification of clinically overt graft rejection.^5-8^ However, no study has analyzed the association between TTV and subclinical rejection.

## MATERIALS AND METHODS

### Patient Cohort and Study Design

All 307 consecutive adult (≥18 y of age) recipients of a kidney allograft transplanted at the Medical University of Vienna, Austria, between December 1, 2016 and December 31, 2018, were screened. Standard of care is described in the Supplement Digital Content (http://links.lww.com/TP/C104). The inclusion criterion was the availability of a protocol biopsy of the kidney graft scheduled 1 year after transplantation. Exclusion criteria were a protocol biopsy earlier than month 9 or later than month 15 posttransplantation, an unstable graft function (defined as a reduction of >20% in the estimated glomerular filtration rate according to the abbreviated Modification of Diet in Renal Disease equation^9^ within the last 3 mo before the biopsy or a urinary protein creatinine ratio >1000 mg/g in combination with an increase of >100% within the last 3 mo before the biopsy), a presumptive polyomavirus nephropathy (defined as a plasma BK viral load ≥1 × 10^4^ copies/mL), and an active participation in interventional trials, respectively. The patients were followed at the outpatient clinic of our center until protocol biopsy or drop out due to a change of posttransplant care center, graft loss, or death. The present study was approved by the local institutional review board (1785/2016) and registered at the German Clinical Trials Registry (DRKS00012335).

### Outcome

The primary outcome was predefined as plasma TTV load in the context of biopsy-proven graft rejection in a protocol biopsy scheduled at month 12 posttransplantation (part of the clinical routine at our center since December 1, 2016). The histologic lesions were categorized following the 2019 Banff Kidney Meeting Report.^10^ Accordingly, borderline changes suspicious for acute T cell-mediated rejection (BL TCMR) were defined by foci of tubulitis (t > 0) with the presence of interstitial inflammation (i > 0) in the primary analysis. For a sensitivity analysis, we used a broader definition of BL TCMR, based on the presence of foci of tubulitis (t > 0) with and without an interstitial inflammation (i). The immunohistochemical assessment of the C4d staining was evaluated on paraffin-embedded sections. The dynamic of chronic lesions in the “month 12” protocol biopsies was assessed by comparing with a “month 3” protocol biopsy or a preimplant biopsy in case of missing “month 3” biopsy (n = 3). The biopsies were assessed by 2 pathologists (J.K. and M.N.) and in case of a disagreement by a third pathologist (H.R.), all blinded to the clinical data.

### TTV Quantification

TTV was quantified prospectively per-protocol in the plasma obtained from peripheral blood at the following predefined time points: before the transplantation and after the transplantation once per week until discharge from the ward, on the first visit at the outpatient clinic, at month 3 after transplantation, and every 3 months thereafter. Earlier studies from our group have described a TTV load <10^6^ copies/mL as a risk factor for clinically overt rejection.^5,8^ Thus, we estimated the days with a TTV load <10^6^ copies/mL between the “month 3” and the “month 12” biopsy for each patient, by summing the days of the intervals with a TTV load <10^6^ copies/mL at the beginning and the end of the interval. The TTV DNA was quantified by a TaqMan real-time polymerase chain reaction, as described previously.^7,11^ Details are described in the Supplement Digital Content (http://links.lww.com/TP/C104). The treating physicians were unaware of the TTV results.

### Statistical Analysis

Generalized linear models for the binominal family were used to estimate the effect size of the association between allograft rejection, according to Banff 2019, presence of histologic lesions, and TTV load at the day of biopsy or days with TTV load <10^6^ copies/mL. The effect size was displayed as a risk ratio (RR) and 95% confidence interval (95% CI). Linear regression was used to quantify the association between the sum of active histologic lesions, the development of chronic allograft injury and TTV load at the time of biopsy or TTV load <10^6^ copies/mL. The effect size was displayed as a coefficient (β) and 95% CI. For sensitivity analysis, the primary cohort was restratified applying a broader definition of BL TCMR based on the presence of any foci of tubulitis (t > 0) with and without interstitial inflammation (i). An additional sensitivity analysis was performed on a cohort including all available “month 12” protocol biopsies. Potential confounders of the effect size of the association of TTV and rejection were assessed using a bivariate analysis. Potential effect modifiers were tested using Mantel-Haenszel strata. A change in the effect size of >10% was defined as significant. For multivariate analysis, the covariables were selected based on their clinical relevance. For the multivariate model, a backward elimination was used, and the “rule of 10” was applied to define the maximum number of the variables allowed to remain in the final model. A *P* value of <0.05 was the predefined limit of significance. Log normal distributed variables were log-transformed (natural log; ln). MS EXCEL 2010 (Microsoft), IBM SPSS Statistics 24.0 (SPSS Inc.), R 4.0.3 (R Core Team), and STATA 15 (STAT Corp.) were applied for the data analysis.

## RESULTS

### Study Cohort

**FIGURE 1.**
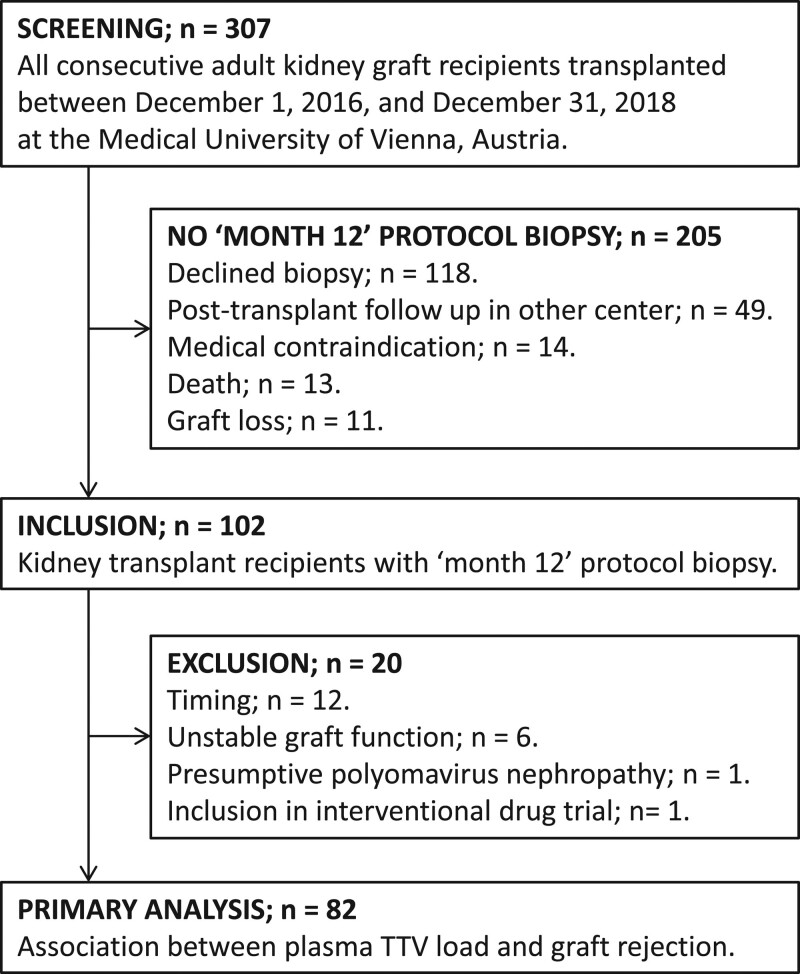
The patient flow of the trial: all 307 consecutive adult recipients of a kidney allograft transplanted at the Medical University of Vienna between December 1, 2016, and December 31, 2018 were screened. One hundred two patients had a protocol biopsy of the kidney graft scheduled 1 y after transplantation. Twenty patients were excluded because of the timing of the protocol biopsy, an unstable graft function, presumptive polyoma virus nephropathy, and active participation in an interventional trial respectively. A total of 82 patients were included in the primary analysis. TTV, torque teno virus.

A total of 307 patients received a kidney transplant at the Medical University of Vienna between December 1, 2016, and December 31, 2018, and 283 had a functioning graft 1 year after transplantation. The study flow is presented in Figure 1. A biopsy was scheduled per-protocol for all kidney graft recipients at month 12 posttransplantation and performed in 102 patients (33%). Eleven recipients experienced a graft loss and 13 died before the biopsy, 118 declined the biopsy, in 14 the biopsy was not performed because of medical reasons, and in 49 because of a change of posttransplant care center. Twenty patients were excluded, 12 because of the timing of their biopsy (biopsy performed later than mo 15 or earlier than mo 9 posttransplantation; Figure 1), 6 because of an unstable graft function (4 had a significant decline in estimated glomerular filtration rate and 2 a significant rise in proteinuria), 1 because of presumptive polyomavirus nephropathy, and 1 because of active participation in an interventional drug trial. A total of 82 stable kidney graft recipients were included in the primary analysis. Baseline characteristics are displayed in Table 1. The median recipient age at transplantation was 53 years, 30% were female, 18% had a history of prior kidney transplantation, 83% received a kidney from a deceased donor, and 10% had preformed donor-specific antibodies (DSAs).

**TABLE 1.** Baseline characteristics of the total study cohort, the cohort with a functioning graft at mo 12 posttransplantation and the cohort selected for analysis of the association between TTV load and subclinical rejection stratified according to biopsy result

	Total cohort (n = 307)	Functioning graft cohort (n = 283)	Biopsy cohort (n = 82)	Rejection (n = 19)	No rejection (n = 63)
Recipient characteristics					
Age (y), median (IQR)	56 (45–64)	55 (45–63)	53 (46–63)	50 (46–61)	53 (47–64)
Female sex	105 (34)	97 (34)	25 (30)	6 (30)	19 (31)
Body mass index^a^	26 (23–29)	26 (23–29)	26 (24–30)	24 (23–27)	26 (24–31)
Cause of end-stage renal disease					
Immunologic	68 (22)	61 (22)	30 (37)	4 (21)	26 (41)
Cystic kidney disease	44 (14)	43 (13)	11 (13)	3 (16)	8 (13)
Diabetes	44 (14)	37 (10)	6 (7)	1 (5)	5 (8)
Hypertension	31 (10)	29 (10)	7 (9)	3 (16)	4 (6)
Hereditary	24 (8)	24 (8)	4 (5)	3 (16)	1 (2)
Other	30 (10)	28 (10)	4 (5)	0	4 (6)
Undefined cause	66 (22)	61 (22)	20 (24)	5 (26)	15 (24)
Time on renal replacement therapy (y), median (IQR)	2.7 (1.5–4.2)	2.6 (1.4–4.1)	2.8 (1.6–4.0)	2.1 (0.7–3.8)	2.9 (1.9–4.2)
Peritoneal dialysis	56 (18)	53 (19)	19 (23)	3 (16)	16 (25)
Donor characteristics					
Deceased donor	260 (85)	239 (84)	68 (83)	14 (74)	54 (86)
Donation after circulatory death	32 (10)	29 (10)	6 (7)	0	6 (10)
Donor age; y, median (IQR)	55 (42–67)	55 (42–66)	55 (44–65)	55 (48–62)	55 (43–67)
Donor female	151 (49)	141 (50)	42 (51)	10 (50)	32 (52)
Transplant characteristics					
History of prior transplantation	59 (19)	54 (19)	15 (18)	5 (25)	10 (16)
ABO-incompatible transplantation	13 (4)	11 (4)	3 (4)	2 (11)	1 (1)
HLA-A/B/DR mismatch (N), median (IQR)	3 (2–4)	3 (2–4)	3 (2–4)	3 (2–4)	3 (2–4)
Preformed donor-specific antibody^b^	25 (8)	22 (8)	8 (10)	1 (5)	7 (11)
Cold ischemia time (h), median (IQR)	12 (8–17)	12 (7–17)	12 (8–18)	10 (7–16)	13 (8–18)
Delayed graft function^c^	71 (23)	54 (19)	16 (20)	2 (11)	14 (21)

Data are presented as number (%) unless otherwise indicated.

There were no significant differences between the total cohort, the cohort with a functioning allograft at mo 12 posttransplant, and the biopsy cohort, respectively. There was no significant difference between patients with and without rejection in the biopsy cohort.

^a^The body mass in kilograms divided by the square of the body height in meters.

^b^All patients had a negative complement-dependent cytotoxicity crossmatch before transplantation.

^c^A delayed graft function was defined by the necessity of >1 renal replacement therapies posttransplant.

IQR, interquartile range; TTV, torque teno virus.

### Graft Function and Biopsy Results

The protocol biopsies of the 82 included kidney transplant recipients were performed at a median of 13 months posttransplant (interquartile range [IQR] 12–14 mo). The median estimated glomerular filtration rate at the time of biopsy was 52 mL/min/1.72m^2^ (IQR 42–66), and the median urinary protein creatinine ratio was 94 mg/g (IQR 67–190). A graft rejection, according to the Banff 2019 Kidney Meeting Report, was diagnosed in 19 of the kidney graft recipients (23%) and 63 showed no rejection. The baseline characteristics, according to rejection status, are displayed in Table 1. In 15 patients, TCMR was described: BL TCMR was diagnosed in 11 and chronic active TCMR in 4 patients. Four patients showed changes compatible with antibody-mediated rejection (AMR; all with detectable DSA at the time of biopsy): active AMR was diagnosed in 1 and chronic active AMR in 2 kidney graft recipients (1 with additional BL TCMR), and C4d staining without evidence of rejection was diagnosed in 1 patient (linear C4d grade 4; ABO compatible transplant).

### TTV Load in the Context of Graft Rejection

All 82 kidney graft recipients included in the primary analysis had detectable TTV at the time of the protocol biopsy with a median of 5 × 10^5^ copies/mL (IQR 8 × 10^4^ to 1 × 10^7^). Patients with graft rejection had a lower TTV load than patients without rejection (median 2 × 10^5^ copies/mL, IQR 3 × 10^3^ to 2 × 10^6^ versus 7 × 10^5^ copies/mL, IQR 1 × 10^5^ to 2 × 10^7^). Each log increase in TTV load decreased the risk for rejection by 9% (RR 0.91, 95% CI, 0.85-0.97; *P* = 0.004). The diagnostic accuracy to detect subclinical rejection by TTV load is displayed in Table 2 and Figure S1 (**SDC**, http://links.lww.com/TP/C104).

**TABLE 2.** Accuracy to detect subclinical rejection diagnosed by protocol biopsy scheduled 12 mo after kidney transplantation by TTV load at the d of biopsy, stratified according to TTV cutoff

TTV (copies/mL)	NPV	PPV	Sensitivity	Specificity
10^4^	0.81	0.43	0.32	0.89
10^5^	0.81	0.33	0.42	0.76
10^6^	0.82	0.27	0.63	0.51
10^7^	0.81	0.25	0.79	0.29

NPV, negative predictive value; PPV, positive predictive value; TTV, torque teno virus.

For sensitivity analysis, the cohort was stratified by applying a broader definition of BL TCMR based on the presence of foci of tubulitis (t > 0) with and without interstitial inflammation (i). Thirteen biopsies were reclassified as BL TCMR, leading to a total number of 32 rejections. A similar effect size of the association between the TTV load and rejection compared to the primary analysis was found (RR 0.92, 95% CI, 0.90-0.94). A second sensitivity analysis was performed on the full set of protocol biopsies (n = 102), including all 20 biopsies excluded from the primary analysis because of timing, graft function, presumptive polyomavirus nephropathy, and active participation in an interventional drug trial. Within this cohort, 4 additional rejection episodes were diagnosed, 2 BL TCMR and 2 chronic active TCMR, meaning that a total number of 23 recipients experienced graft rejection. A similar effect size of the association between the TTV load and rejection compared to the primary analysis was found (RR 0.94, 95% CI, 0.87-1.01).

Subgroup analysis restricted to the patients with an AMR (n = 4) revealed an effect size of the association between rejection and TTV load comparable to the primary analysis, including all rejections (RR 0.92, 95% CI, 0.81-1.07). Similar results were obtained in a further subgroup analysis restricted to TCMR (n = 15; RR 0.91, 95% CI, 0.81-1.03). The largest effect size of the association between rejection and TTV load was obtained in the final subgroup analysis restricted to AMR and TCMR excluding BL TCMR (n = 8; RR 0.85, 95% CI, 0.76-0.95).

To test whether the TTV load was independently associated with rejection, predefined potential confounders were analyzed, and the adjusted RR was calculated (Table 3). None of the tested variables, including the recipient age and sex, preformed DSA, history of prior transplantation or ABO-incompatible transplantation, HLA mismatch, prior rejection episodes, posttransplant DSA, a BK viremia, the graft function, the immunosuppression at the day of the protocol biopsy, and the timing of the protocol biopsy, respectively, changed the effect size of the association between TTV level and rejection significantly. Moreover, the recipient age and sex were no significant effect modifiers on the association between the TTV level and rejection.

**TABLE 3.** Unadjusted and adjusted effect size of the association between TTV load at the time of biopsy and subclinical rejection detected in 12 mo protocol biopsies (RR per each log increase in TTV load)

Method	Covariables	Risk ratio	95% Confidence interval
Unadjusted		0.91	0.85-0.97
Adjusted	Recipient age at transplantation	0.92	0.86-0.98
	>53 y^a^	0.90	0.86-0.94
	≤53 y^a^	0.95	0.82-1.10
	Recipient sex	0.91	0.84-0.97
	Female	0.93	0.80-1.08
	Male	0.89	0.85-0.94
	ABO-incompatible transplantation	0.90	0.84-0.97
	Preformed donor-specific antibodies	0.91	0.85-0.97
	History of prior transplantation	0.92	0.86-0.97
	HLA mismatch	0.91	0.84-0.97
	Time between kidney transplantation and protocol biopsy	0.91	0.85-0.98
	Posttransplant donor-specific antibodies^b^	0.92	0.88-0.96
	Posttransplant BK polyoma viremia^c^	0.92	0.86-1.00
	BK polyoma viremia at protocol biopsy^c^	0.93	0.85-1.00
	Graft rejection	0.90	0.87-0.94
	Tacrolimus based immunosuppression^e^	0.90	0.84-0.95
	Tacrolimus trough level^e^	0.92	0.86-0.97
	Full dose mycophenolic acid^e,f^	0.91	0.87-0.94
	Triple immunosuppression^e^	0.90	0.85-0.95
	Estimated glomerular filtration rate^e^	0.91	0.85-0.98
Final model	Recipient age and sex	0.92	0.86-0.98

^a^Median age.

^b^Number of posttransplant donor-specific antibodies (n = 21).

^c^Any BK polyoma viremia detected by a polymerase chain reaction (n = 21).

^d^Any biopsy-proven graft rejection before the protocol biopsy (n = 24).

^e^At the time of the protocol biopsy.

^f^2000 mg mycophenolate mofetil or 1440 mg mycophenolic acid per d.

RR, risk ratio; TTV, torque teno virus.

### TTV Load in the Context of Histologic Single Lesions

In 63% of the patients (n = 51), at least 1 active single lesion including g, v, t, i, ti, ptc, iIFTA, tIFTA, or C4d was detected in the “month 12” protocol biopsy with a median lesion sum of 3 (IQR 0–6) per individual. The sum of active lesions was associated with the TTV load (coefficient −0.05, 95% CI, −0.10 to −0.01; *P*= 0.02). Kidney graft recipients with active lesions had a lower TTV load at the time of biopsy than patients without such lesions (median 4 × 10^5^ copies/mL, IQR 2 × 10^4^ to 7 × 10^6^ versus median 2 × 10^6^ copies/mL, IQR 2 × 10^5^ to 5 × 10^7^). TTV load stratified according to histologic lesion is displayed in Figure S2 (**SDC**, http://links.lww.com/TP/C104). The risk for the presence of active lesions decreased by 4% with each increase in the TTV log level (RR 0.96, 95% CI, 0.95-0.97; *P* < 0.001). The effect size of the association between the presence of each active histologic single lesion and the TTV load at the time of biopsy is displayed in Table 4.

**TABLE 4.** Effect size of the association between the presence of active histologic lesions at the 12 mo protocol biopsy and the TTV load at the time of biopsy (RR per each log increase in TTV load)

Lesion^a^	Biopsies with analyzable compartment (n)	Biopsies with detectable lesion (n; %)	Risk ratio	95% Confidence interval
i	79	33 (42)	0.99	0.93-1.04
t	80	28 (35)	0.98	0.92-1.04
v	80	0		
g	81	7 (9)	0.89	0.77-1.03
ptc	79	8 (10)	0.91	0.79-1.05
C4d	79	3 (4)	0.71	0.60-0.83
ti	81	47 (58)	0.99	0.95-1.03
iIFTA	81	46 (57)	0.98	0.94-1.02
tIFTA	81	35 (43)	0.98	0.92-1.03

^a^Histologic lesions were scored according to the 2019 Banff Kidney Meeting Report (yes vs no).^10^

RR, risk ratio; TTV, torque teno virus.

Almost all patients (n = 79, 96%) had detectable chronic lesions including cg, cv, ci, ct, ah, and ptcml in the “month 12” protocol biopsy with a median lesion sum of 4 (IQR 3–6) per individual. The dynamic of the chronic lesions was analyzed in the context of a protocol biopsy scheduled 3 months posttransplantation (median 96 d posttransplantation, IQR 88–108). The median sum of the increase in chronic lesions from the “month 3” to the “month 12” biopsy was 1 per individual (IQR 0–3). To assess the association between a low TTV load and the devolvement of chronic graft damage over time we calculated the days with a TTV load <10^6^ copies/mL between the “month 3” and the “month 12” biopsy for each patient (median 67, IQR 0–157; for the 3 patients with missing mo 3 biopsy the preimplant biopsy was chosen and TTV load accessed from mo 3). The increase in chronic lesions was associated with the days with a TTV load <10^6^ copies/mL within the same period of time (coefficient 0.07, 95% CI, 0.01-0.14; *P* = 0.02). The diagnostic accuracy to detect development of chronic lesions by days with a TTV load below 10^6^ copies/mL is displayed in Table 5 and Figure S3 (**SDC**, http://links.lww.com/TP/C104). The effect size of the association between the development of each chronic histologic single lesion from months 3 to 12 and the days with a TTV load below 10^6^ copies/mL within the same period is displayed in Table 6. Days with TTV load below 10^6^ copies/mL stratified according to histologic lesion is displayed in Figure S4 (**SDC**, http://links.lww.com/TP/C104).

**TABLE 5.** Accuracy to detect de novo chronic histologic lesions (development of any cg, cv, ci, ct, ah, and ptcml comparing the protocol biopsy scheduled at mo 3 and at mo 12 posttransplantation) by the number of d with a TTV load <10^6^ copies/mL (cumulative during the period between the 2 biopsies). Stratification was performed according to time intervals with a TTV load below 10^6^ copies/mL

Days	NPV	PPV	Sensitivity	Specificity
30	0.48	0.82	0.78	0.55
90	0.34	0.82	0.46	0.73
180	0.28	0.75	0.22	0.82
270	0.26	0.67	0.10	0.86

NPV, negative predictive value; PPV, positive predictive value; TTV, torque teno virus.

**TABLE 6.** Effect size of the association between the development of changes in chronic histologic lesions from months 3 to 12 and the number of d with a TTV load <10^6^ copies/mL within the same period (RR per each log increase in d)

Lesion^a^	Biopsies with analyzable compartment (n)	Biopsies with detectable lesion, n (%)	Risk ratio	95% Confidence interval
ci	78	35 (45)	1.11	0.97-1.27
ct	81	26 (27)	1.13	0.95-1.34
cv	76	20 (26)	1.09	0.90-1.32
cg	81	3 (4)	0.97	0.59-1.59
ptclm	81	12 (15)	1.08	0.83-1.39
ah	81	16 (13)	1.13	0.90-1.43
C4d	78	2 (3)	1.97	0.41-9.34
iIFTA	80	41 (52)	1.19	1.04-1.35
tIFTA	80	26 (33)	1.31	1.05-1.62

^a^Histologic lesions were scored according to the 2019 Banff Kidney Meeting Report (yes vs no).^10^

RR, risk ratio; TTV, torque teno virus.

## DISCUSSION

The major finding of this trial was the establishment of a robust association between allograft rejection and TTV load in clinically stable patients 1 year after kidney transplantation. Subclinical alloreactivity and the development of chronic graft injury were associated with a low TTV load suggesting improved virus control due to a less suppressed immune system. Early recognition of insufficient immunosuppression might offer a therapeutic window to optimize immunosuppressive drugs. Such personalized adaption of the immunosuppression might reduce alloreactivity and subsequent graft injury and thus provides a valuable strategy with the potential of being tested in a randomized controlled setting.

The primary analysis of the present trial was restricted to stable patients. However, a comparable effect size of the association between TTV load and rejection was calculated for the total unselected cohort. Therefore selection bias is unlikely. The primary outcome included BL TCMR, as recent data suggested a clinical significance of such pattern in biopsies taken 1 year after kidney transplantation.^12,13^ Reanalysis of the cohort, applying a more sensitive threshold for BL TCMR, described a similar effect size of the association between TTV load and graft rejection. This finding links tubulitis without interstitial inflammation with low immunosuppression. Indeed, a recent report suggests tubulitis without interstitial inflammation as a risk factor for acute rejection and graft loss.^14^ The largest effect size of the association between the TTV load and histologic changes was detected in a compound endpoint, including all the single lesions suggestive for active alloreactivity and a subgroup analysis restricted to AMR and TCMR excluding BL TCMR. These findings suggest that TTV load best reflects the total amount of active alloreactivity and the more severe rejection episodes, respectively. We describe a comparable effect size of the association between TCMR, AMR, and TTV load, suggesting TTV load reflects the overall immune function, rather than an isolated T-cellular specific immune response. Statistical tests were only performed for the primary outcome. Therefore data concerning subgroup analysis and secondary outcomes including histologic lesions have to be interpreted with caution and might only be hypothesis generating. Of note our dataset does not include acute TCMR I or more severe cases, limiting the generalizability of our results for such rejection classes.

Our results describe only a modest accuracy of TTV load to detect subclinical rejection. In this respect, our report is in line with published work on clinically overt rejection.^6,8^ However, for TTV loads <10^6^ copies/mL increasing specificity for the prediction of subclinical rejection was calculated from our data. Moreover, TTV load in patients experiencing rejection or histologic lesions suggestive of alloreactivity was 10^5^ copies/mL compared to 10^6^ copies/mL in patients without rejection and without such lesions, respectively. The development of chronic graft injury was associated with the number of days with a TTV load <10^6^ copies/mL, a cutoff that has already been suggested as a critical risk factor for clinically overt rejection in earlier studies from our group.^5,8^ However, only a modest accuracy for the number of days with a TTV load <10^6^ copies/mL to predict the development of chronic lesions was calculated from our present data. Of note, specificity to detect chronic changes rises if a TTV load below 10^6^ copies/mL was detected for >90 days. In this respect, it is important to note, that TTV was measured every 3 months per protocol. Therefore our findings might suggest, that at least 2 measurements of a TTV load <10^6^ copies/mL or a TTV <10^6^ copies/mL during a period of >3 months are needed to increase the risk for chronic graft injury. Taken together, diagnostic accuracy of TTV to predict subclinical graft injury is only modest. However, our data suggest TTV loads <10^6^ copies/mL as a useful target to be tested in a larger multicenter prospective setting or interventional trials.

A limitation of the study is the high rate of loss to follow-up. Of note, baseline characteristics were similar in the total cohort, the cohort with a functioning graft 12 months posttransplantation and the cohort included in the primary analysis. Therefore selection bias is unlikely. Another limitation is the sample size and the single-center design. Differences in baseline characteristics and posttransplantation care might reduce generalizability of our results. Intercenter variability of TTV PCR might further complicate comparability of TTV loads reported in this article. In this respect, it is important to note that a standardization process for the TTV quantification was initiated in 2018 with high accuracy^15^ and a commercial TTV assay was introduced in 2017.^16^ Such tools will facilitate prospective, large scale multicenter studies necessary to test the findings from our report. Finally, our study design cannot prove causality between TTV and immunosuppression or clinical relevance of the association between TTV load and subclinical rejection. Interventional trials based on TTV load are needed to analyze the value of TTV-guided drug dosing (eg, adaption of the tacrolimus dose according to a predefined range of TTV load in the plasma).

This trial is the first to associate TTV load and alloreactivity in stable kidney transplant recipients. Multicenter and interventional studies are needed to test the value of TTV quantification for the guidance of immunosuppression.

## ACKNOWLEDGMENTS

The authors would like to thank Gabriele Rath, Habiba Ahmed, and Luis Naar for their excellent support in clinical data collection.

## Supplementary Material


