# Supplementary material for: Torque Teno Virus Load Is Associated With Subclinical Alloreactivity in Kidney Transplant Recipients: A Prospective Observational Trial
Source: Transplantation. 2021 Aug 19;105(9):2112-2118. doi: 10.1097/TP.0000000000003619 (PMC8376270; doi:10.1097/TP.0000000000003619)
Supplement: Supplementary file 1 [file tp-105-2112-s001.pdf]

SUPPLEMENTARY DIGITAL CONTENT

SUPPLEMENTARY MATHERIALS AND METHODS

Standard of care for immunosuppression and prophylaxis

All patients received a triple immunosuppressive treatment with tacrolimus (target for month 3 to 12 post-transplantation: 6 to 8 ng/mL trough level), mycophenolic acid (target 2g, daily fixed dose), and corticosteroids. All HLA-compatible patients received an induction therapy with an interleukin-2 receptor blockade (Simulect; Novartis AG, Switzerland). Recipients of an HLA-incompatible kidney, defined by the presence of a preformed donor-specific antibody (DSA), underwent IgG immunoapheresis and received an anti-thymocyte globulin (Grafalon; Neovii, Switzerland) according to a local protocol.[1] Recipients of an ABO-incompatible kidney underwent an ABO blood group antigen-specific immunoadsorption (Glycosorb; Glycorex Transplantation AB, Sweden). All patients received prophylaxis with trimethoprim-sulfamethoxazole for 6 to 12 months after transplantation and valganciclovir for 3 months in case of a cytomegalovirus IgG-negative recipient of a cytomegalovirus IgG-positive organ, in case of Epstein-Barr virus IgG-negativity of the recipient and after treatment with depleting antibodies and an IgG immunoapheresis.

Standard of care for rejection

T-cell mediated rejection type I and II and borderline changes suspicious for acute T-cell mediated rejection were treated with the equivalent of 100mg dexamethasone for 3 days and TCMR rejection type III or steroid refractory TCMR with 3 mg/kg anti-thymocyte globulin (Grafalon, Neovii, Switzerland) for 10 days. Antibody-mediated rejection was treated with immunoadsorption[2] and in case of refractory rejection with additional membrane filtration.[3]

Routine monitoring for donor-specific antibodies

Donor-specific antibodies were routinely assessed at months 3 and 12 post-transplantation by single-antigen flow bead testing (LABScreen Single Antigen assays; One Lambda). Before testing, patient sera were heat inactivated (30 minutes, 56°C). Thresholds were set at a median fluorescence intensity >1000. Donor specificity of HLA reactivity was determined according to high-resolution donor/recipient HLA typing (HLA-A, -B, -Cw, -DR, -DQ, and/or -DP).

Routine screening for viral disease

Screening for BK polyomavirus after transplantation was performed by polymerase chain reaction from peripheral blood once per week until discharge from the ward, on the first visit at the outpatient clinic, on month 3 after transplantation, every 3 months thereafter.

Torque Teno virus quantification

TTV DNA was extracted from 200 µL of plasma using the NucliSENS easyMAG platform (bioMeriéux, France), as recommended by the manufacturer. The TTV DNA was quantitated by a TaqMan real-time polymerase chain reaction, as described previously.[4,5] Reactions were performed in a volume of 25 µL using 2 × a TaqMan Universal Master Mix, containing 5 µL of the extracted DNA, 400 nM of the primer, and 80 nM of the probe. The thermal cycling was started for 3 minutes at 50°C, followed by 10 minutes at 95°C, and then by 45 cycles at 95°C for 15 seconds, at 55°C for 30 seconds, and at 72°C for 30 seconds, using the CFX96 Real-time System (Bio-Rad, USA). Linear range was from $1x10^3$ to $1x10^{11}$ c/mL and limit of detection was $1x10^3$ c/mL.

SUPPLEMENTARY FIGURE LEGEND

FIGURE S1. The receiver operating curve to predict subclinical rejection by TTV load is plotted and the corresponding area under the curve is calculated (0.617, 95%CI 0.462 - 0.771). Sensitivity is displayed on the y-axis and 1-Specificity on the x-axis.

FIGURE S2. Torque Teno virus load (ln copies per mL) at the time of biopsy is represented for individual patients stratified according to the presence of acute lesions (A to H; abbreviation according to Banff) in the month 12 protocol biopsy. TTV load is displayed in box-plots combining all measurements according to the category on the x-axis. The box represents the distance between the first and third quartiles. The horizontal line in the box represents the median. The upper whisker extends to the largest value no further than 1.5 * inter-quartile range. The lower whisker extends to the smallest value at most 1.5 * IQR. Data beyond the end of the whiskers are plotted individually.

FIGURE S3. The receiver operating curve to predict development of chronic lesions by days with a TTV load below 10^6 c/mL load is plotted and the corresponding area under the curve is calculated (0.616, 95%CI 0.460 - 0.772). Sensitivity is displayed on the y-axis and 1-Specificity on the x-axis.

FIGURE S4. Days of Torque Teno virus load below 10^6 copies per mL (ln) between the month 3 and the month 12 protocol biopsy are represented for individual patients stratified according to the development of chronic lesions in the same period (A to I; abbreviation according to Banff). Days are displayed in box-plots combining all measurements according to the category on the x-axis. The box represents the distance between the first and third

quartiles. The horizontal line in the box represents the median. The upper whisker extends to the largest value no further than 1.5 * inter-quartile range. The lower whisker extends to the smallest value at most 1.5 * IQR. Data beyond the end of the whiskers are plotted individually.

Refrences

1. Bartel G, Wahrmann M, Regele H, et al. Peritransplant immunoadsorption for positive crossmatch deceased donor kidney transplantation. *Am J Transplant*. 2010;10(9):2033-2042.

2. Bohmig GA, Wahrmann M, Regele H, et al. Immunoadsorption in severe C4d-positive acute kidney allograft rejection: a randomized controlled trial. *Am J Transplant*. 2007;7(1):117-121.

3. Doberer K, Bond G, Klager J, et al. Immunoadsorption Combined with Membrane Filtration to Counteract Early Treatment-Refractory Antibody-Mediated Rejection. *Blood Purif*. 2020:1-10.

4. Maggi F, Pifferi M, Fornai C, et al. TT virus in the nasal secretions of children with acute respiratory diseases: relations to viremia and disease severity. *J Virol*. 2003;77(4):2418-2425.

5. Schiemann M, Puchhammer-Stockl E, Eskandary F, et al. Torque Teno Virus Load-Inverse Association With Antibody-Mediated Rejection After Kidney Transplantation. *Transplantation*. 2017;101(2):360-367.

FIGURE S1

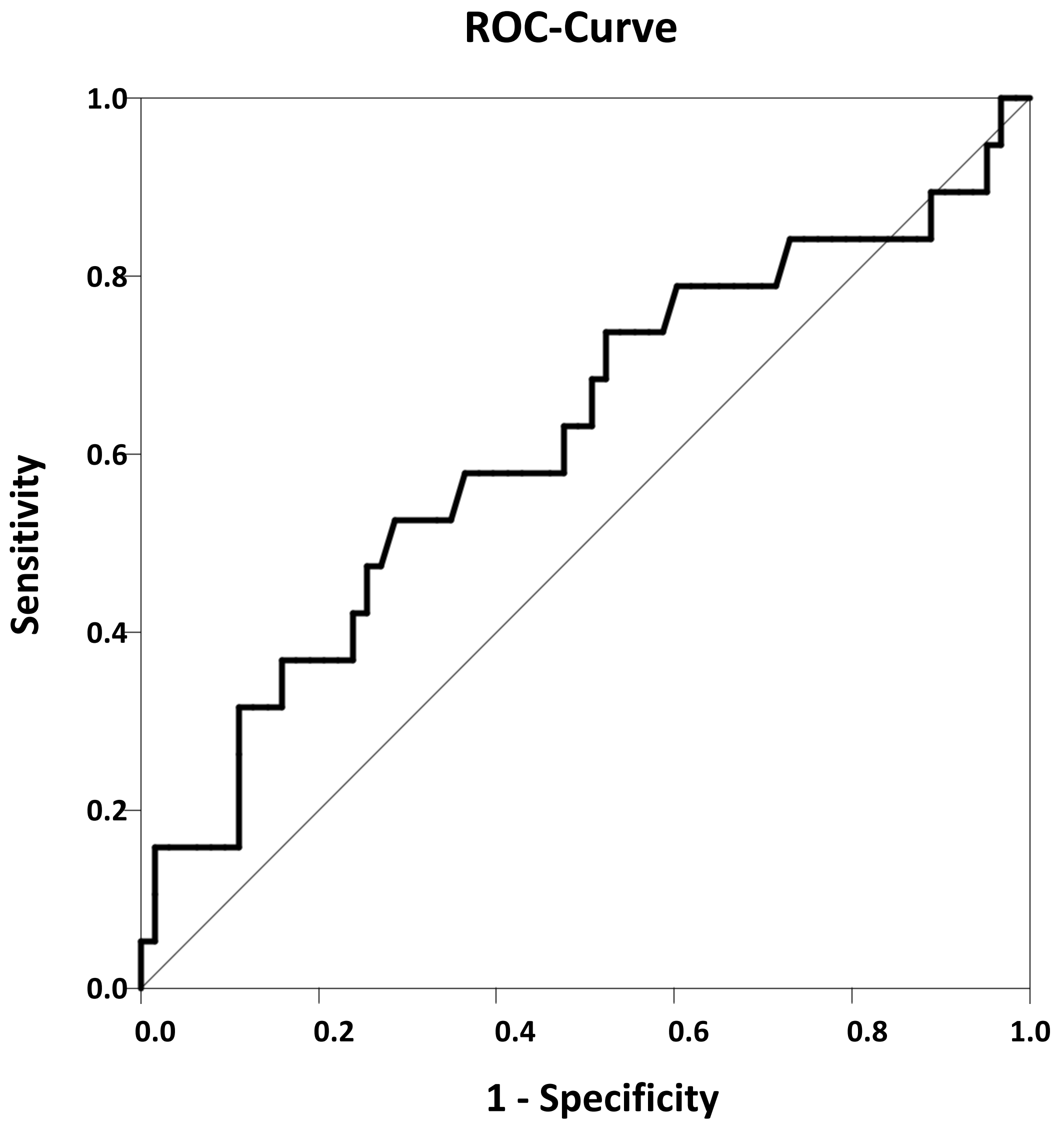
ROC-Curve
1.0
0.8
0.6
0.4
0.2
0.0
Sensitivity
0.0
0.2
0.4
0.6
0.8
1.0
1 - Specificity

FIGURE S2

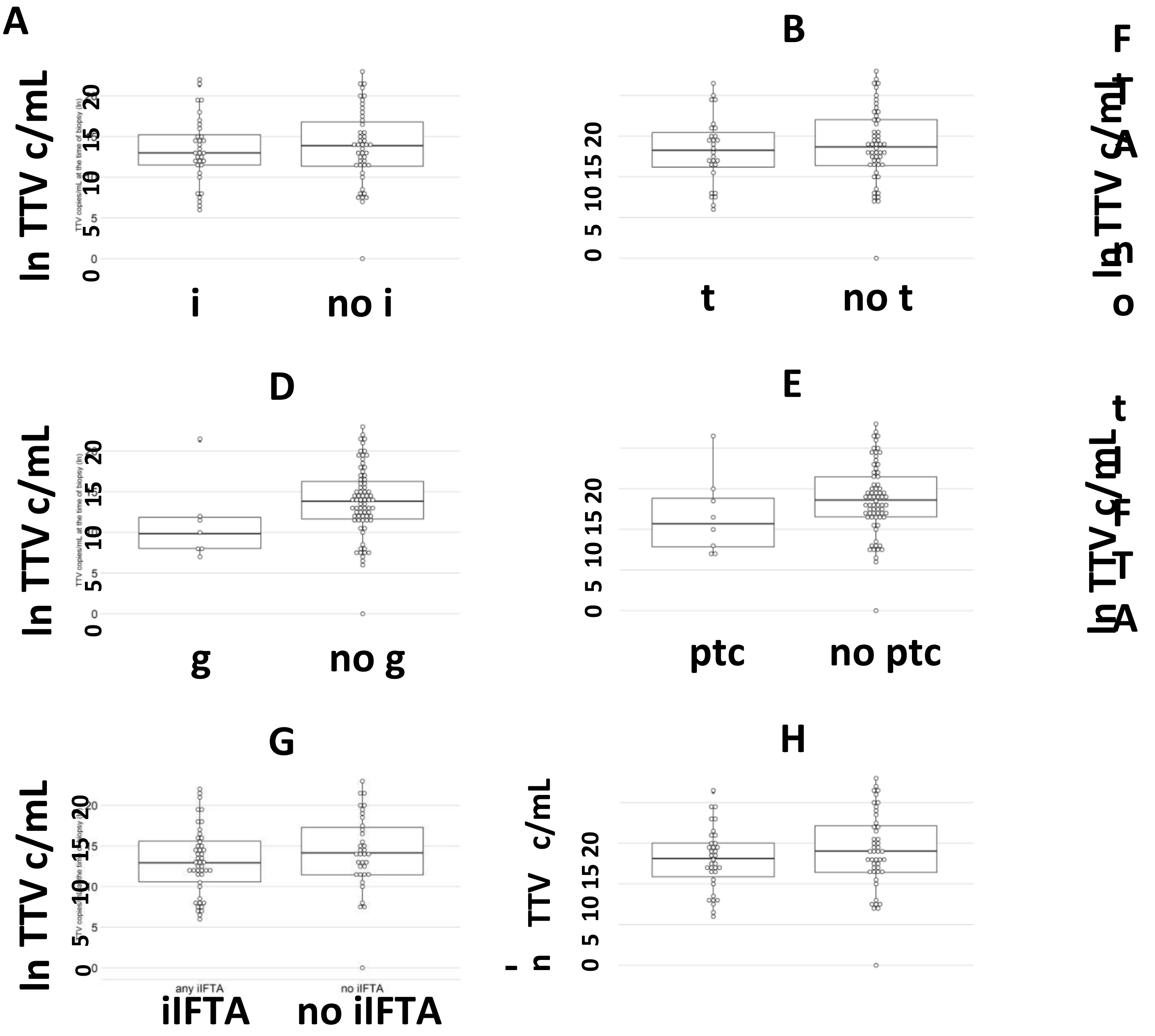

A
B
D
E
G
H
ln TTV c/mL
0 5 10 15 20
i
no i
t
no t
g
no g
ptc
no ptc
any iIFTA
no iIFTA
iIFTA
no iIFTA

C

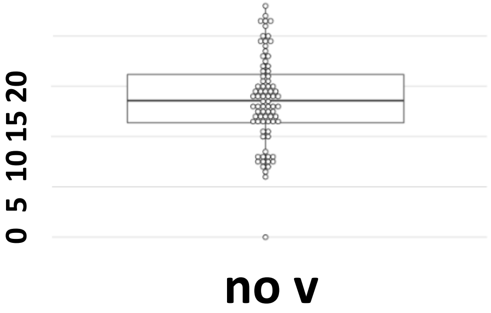

F

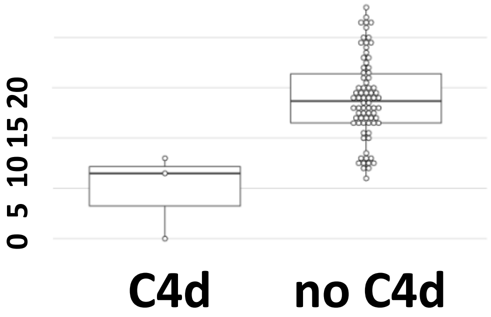

FIGURE S3

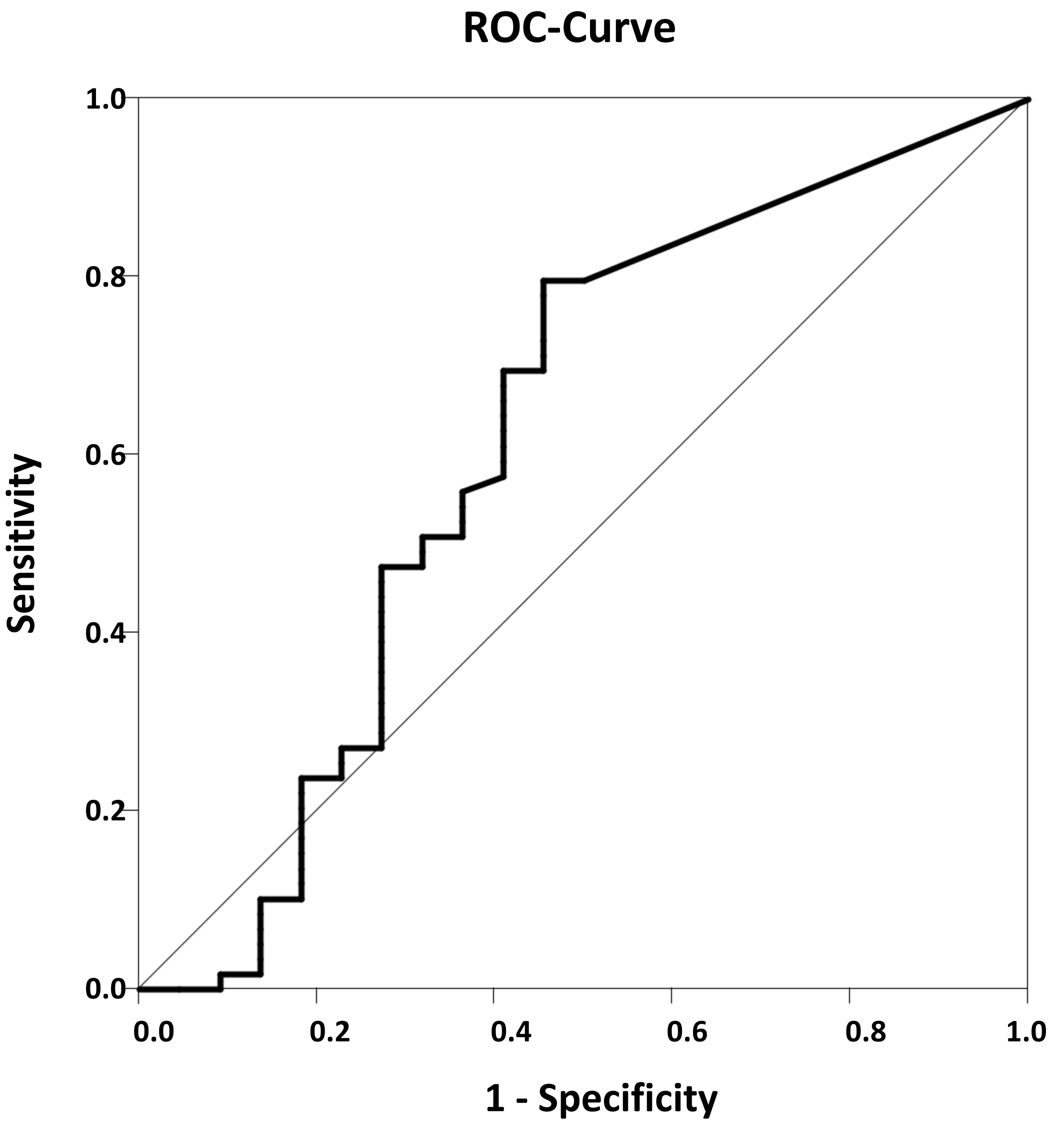
ROC-Curve
1.0
0.8
0.6
0.4
0.2
0.0
Sensitivity
0.0
0.2
0.4
0.6
0.8
1.0
1 - Specificity

FIGURE S4

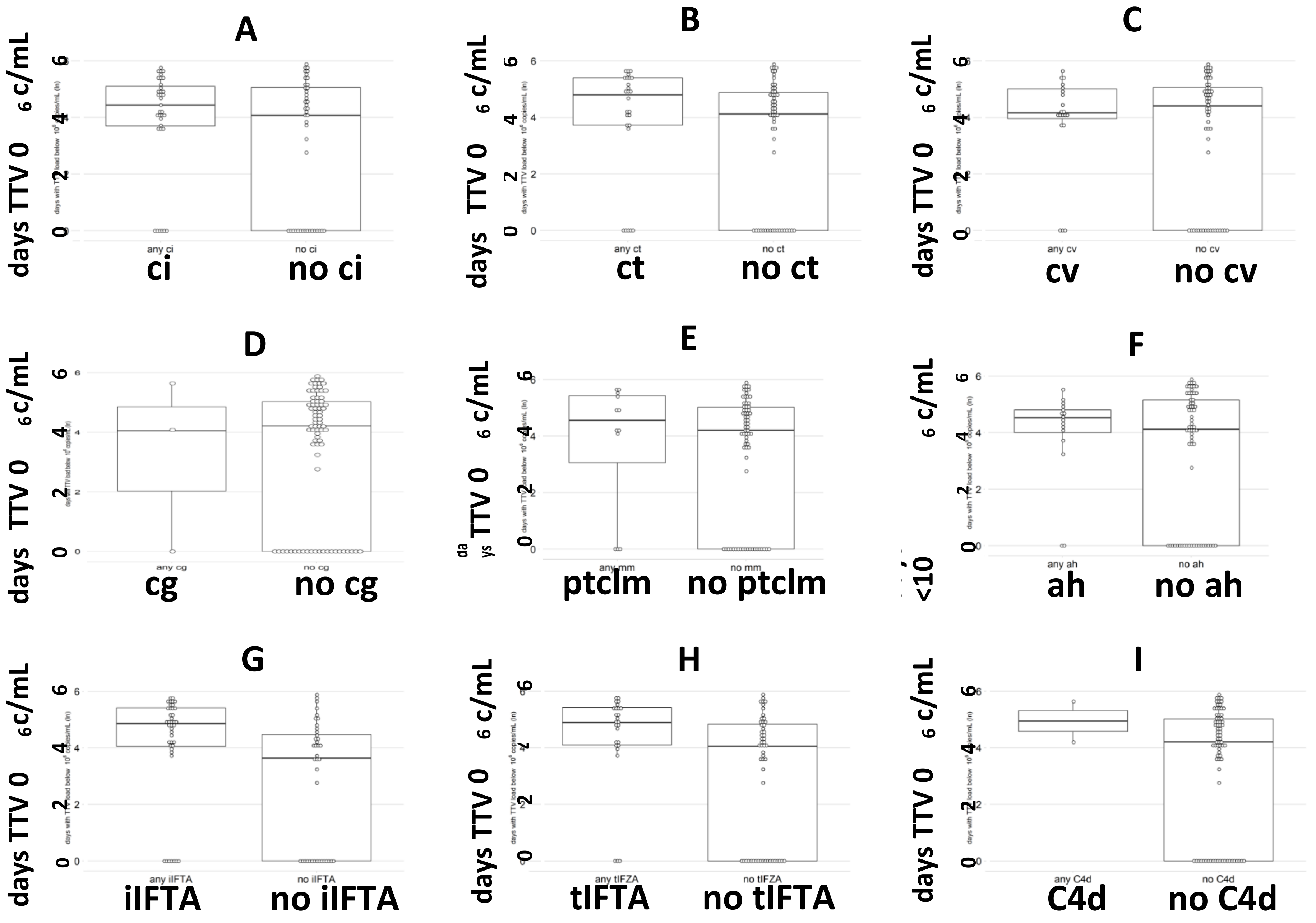
A
B
C
D
E
F
G
H
I
days TTV 0 6 c/mL
ci
no ci
ct
no ct
cv
no cv
cg
no cg
ptclm
no ptclm
ah
no ah
iIFTA
no iIFTA
tIFTA
no tIFTA
C4d
no C4d
<10
